# A Differential Evolution-Based Optimized Ensemble for Balanced and Imbalanced Medical Datasets

**DOI:** 10.12688/f1000research.169456.2

**Published:** 2026-01-27

**Authors:** Surajit Das, Samaleswari P. Nayak, Biswajit Sahoo, Satyananda Champati Rai

**Affiliations:** 1 School of Computer Engineering, Kalinga Institute of Industrial Technology, Bhubaneswar, Odisha, 751024, India; 2 Department of Computer Science and Engineering, Silicon University, Bhubaneswar, Odisha, 751024, India

**Keywords:** Ensemble Learning, Differential Evolution, Class Imbalance, AUC Optimization, SMOTE, ADASYN.

## Abstract

**Background:**

Class imbalance is a frequent and severe problem in medical datasets, where instances from the minority class are usually high risk or disease positive. Most traditional classifiers suffer from a biasness towards the majority class, resulting in a poor detection rate of the minority class and, therefore, decreased confidence in prediction systems in medical applications.

**Methods:**

In this paper, we present an optimized ensemble by differential evolution (OEDE), a novel ensemble learning framework, to address this problem. OEDE harmonizes three dissimilar base learners (Logistic Regression, Random Forest, and XGBoost) and trains each using class-balancing techniques. Next, the model utilized Differential Evolution (DE) to discover the most appropriate ensemble weights to maximize the area under the ROC curve (AUC) on a validation dataset.

**Result:**

We conducted experiments on four real-world medical datasets, whose imbalance ratios vary from 1.89 to 14.6, using OEDE in the original, SMOTE, and ADASYN balanced conditions. Experimental results demonstrate substantial performance gain of OEDE on the challenging Thoracic dataset, achieving a 70.08% AUC, outperforming the standard Random Forest (50.82%) and AdaBoost (47.15%) baselines by over 19%. Additionally, on the Cervical Cancer dataset, the model achieved a peak AUC of 97.89%. The results indicate that the proposed OEDE consistently outperforms or is competitive with traditional ensemble models in terms of AUC, F1-score, and Recall. ROC curve analysis also approved the OEDE’s superior discriminative capabilities.

**Conclusion:**

The proposed OEDE framework effectively improves minority class detection in imbalance medical datasets. Its robust and flexible design makes it a promising tool for healthcare risk prediction tasks where minority class groups need to be well identified.

## 1. Introduction

Machine learning has become increasingly popular in medical and healthcare services in recent years because it can be employed to analyze multidimensional datasets and detect subtle patterns that may not be detectable using traditional standard statistical methods. ^1, 2^ Disease diagnosis, estimation of survival, and risks using machine learning models are becoming more common in assisting clinical decision-making. However, one of the most common and serious issues in medical datasets is class imbalance, in which an individual class label with a smaller number of instances occurs more infrequently than the others. ^3^ As a result, traditional models may be biased in favour of the majority class, which may lead to a loss of sensitivity and misclassification of rare but significant minorities.

Many real-world medical datasets, such as cancer prediction, surgical outcomes, and disease screening, suffer from moderate to severe class imbalance. Models trained on imbalanced data often achieve high overall accuracy because they only predict the majority class but may have a poor generalization to the minority class. This is a problem in healthcare, where a missed positive result can be catastrophic. People face this challenge with traditional resampling methods such as SMOTE ^4^ and ADASYN ^5^ and ensemble methods ^6^ such as AdaBoost (AB), CatBoost (CB), Gradient Boost (GB), XGBoost (XGB), Random Forest (RF), Balanced Random Forest (BRF), LightGBM (LGBM), Easy Ensemble (EE), and Extra Trees (ET). While previous research has shown increased classification capabilities, they are still constrained by static or heuristic based ensemble integration algorithms such as simple averaging ^7^ or static weights, ^8^ that lack the freedom to adaptive weight initialization of the base learners. Additionally existing approaches typically rely on minimizing surrogate loss functions ^9^^,^^10^ rather than directly maximizing non-differentiable, clinically relevant evaluation matrices such as AUC, which is critical for robust performance evaluation on imbalanced medical dataset.

For imbalance classification, there is still a gap in ensemble model design, where most models use static or heuristic weights for the base models. Hence, an adaptive ensemble model is needed that incorporates class balance into training and learns to correctly weight the outputs of the base learners. To address these goals, we proposed a novel ensemble model called Optimized Ensemble by Differential Evolution (OEDE) using an adaptive weighted ensemble, where the optimal weights are determined by Differential Evolution (DE). The main contributions of this study are as follows:

• A novel ensemble architecture that employs Differential Evolution fro direct maximization of non-differentiable matrix like AUC to bypass the gradient-based meta learner’s limitations.
• A dynamic weight evolution strategy that adapts to dataset imbalance prioritizing base learners that effectively capture minority classes.
• Empirical evidences show robustness of OEDE across different imbalance ratios (1.89 – 14.6), outperforming traditional ensembles in high-imbalance scenarios.

## 2. Related work

Dey and Pratap ^11^ studied different oversampling techniques, such as SMOTE, Borderline-SMOTE, and ADASYN, on different statistical models, such as SVM, KNN, GNB, DT, and RF, concluding that RF combined with SMOTE outperforms others. T.-C.T. Chen et al. ^12^ used an ensemble approach for the classification of diabetes, where DNN performed the classification, and a modified RF was used to explain the results of the proposed model. MARTÍNEZ-VELASCO et al. ^13^ have used both oversampling and under-sampling along with 8 different ML models and concluded that the balanced bagging and balanced RF (BRF) beat every other setup, even without balancing the dataset. To address this class imbalance problem, Agyemang et al. ^14^ used several oversampling techniques such as Random Oversampling (RO), SMOTE, SMOTE-Tomek, and ADASYN, along with ML models such as K-Nearest Neighbour (KNN), Support Vector Machine (SVM), Logistic Regression (LR), Random Forest (RF), and Decision Tree (DT), and concluded that RO-SVM gave the best result. Abayomi-Alli et al. ^15^ proposed a 2-phase ensemble model combining DNN with 15 other ML models (ExtraTrees, SVM, RBF, etc.) for COVID-19 classification, showing that the DNN-ExtraTrees ensemble performed better than the other combinations.

Elgendy et al. ^16^ used a stacking-based ensemble of seven base models for diabetes prediction and concluded that the stacked multilayer perceptron (MLP) provides the highest accuracy. Dutta et al. ^7^ applied weighted average ensemble strategies with GNB, BNB, RF, DT, XGB, and LGB and concluded that the DT+RF+XGB+LGB pair achieves 73.5% accuracy, which is the highest among all other pairs. Alzakari et al. ^17^ proposed a two-stage ensemble combining XGBoost and Bi-LSTM, where XGBoost performs feature selection and early classification and Bi-LSTM performs second stage classification and pattern recognition. Das et al. ^18^ conducted studies of different ML models in different class-imbalanced datasets and concluded that RF performs remarkably well in both balanced and imbalanced datasets. Senthilvadivu et al. ^19^ used RF and XGB for decision making in ICU patients and showed that XGB performs better than RF.

For the prediction of heart disease, Abdellatif et al. ^8^ proposed a weighted random forest ensemble model, used along with an infinite feature selection strategy, and concluded that the proposed model performed better than the SMOTE-RF combination. Yalin et al. ^20^ proposed the XGBoost-BLR method for the classification of diabetes, where XGBoost is used to transform selected features into higher dimensions, and binary logistic regression (BLR) was used for modelling the higher dimensional data. Abnoosian el al. ^21^ used a normalized weighted ensemble to aggregate the results of six different ML models for the classification of diabetes, showing that the proposed ensemble model performed better than the individual base models. Liu et al. ^9^ used bagging to overcome the problem of an imbalanced dataset, where LR was used for feature selection, and SVM was used as a weak classifier. For heart disease prediction, Masruriyah et al. ^22^ used SMOTE and ADASYN along with four ML models and concluded that the oversampling techniques cause a reduction in the accuracy of the model.

Pablo et al. ^23^ performed an analysis of different ML models and sampling techniques on the COVID-19 dataset and concluded that MLP stood out strongly in all combinations, whereas SVM gave lower performance in all combinations. To classify COVID-19, Chowdhury et al. ^24^ proposed a two-step ensemble pipeline with four different ML models, where the results of KNN, SMV, and XGB were passed through RF for final prediction, and concluded that the proposed model outperformed the existing models. Prithula et al. ^25^ proposed a stacking ensemble with ET, RF, and CB as base models and GB as a meta-learner, and concluded that CB outperforms the proposed model. A study by Chowdhury et al. ^26^ Performance analysis of different ML models was performed in the original dataset, and after the dataset was balanced using oversampling, under-sampling, and hybrid-sampling techniques, it was shown that the ML models performed better in the original dataset, except that there was a minor improvement in recall after the dataset was balanced. To handle the imbalanced dataset, Mienye and Sun ^10^ proposed four cost-effective ML models by tuning the hyperparameters and concluded that cost-sensitive XGB performs better than the other models.

In summary (as shown in Table 1), various strategies have been explored to address the class imbalance problem. Oversampling methods such as SMOTE and ADASYN, under-sampling techniques such as RUS and ENN, and hybrid sampling techniques such as SMOTE-ENN and SMOTE-Tomek have been widely used to improve the performance of the models. Many studies have shown that ensemble models can improve the performance by assembling multiple base models. In fact, models such as RF and XGBoost are highly effective across different healthcare applications, as shown in different studies. Overall, the literature suggests that there is no universal solution, and that the selection of the technique is typically based on the nature of the dataset.

**Table 1.** Literature review.

Author	Disease	Models	Is the original dataset imbalanced	Imbalance handle strategy	Observation
Dey and Pratap ^11^	Diabetes and Breast Cancer	SVM, KNN, GNB, DT, RF	Yes	SMOTE, Borderline-SMOTE, ADASYN	RF in combination with SMOTE outperforms others.
Chen et al. ^12^	Diabetes	DNN, RF	Yes	-	RF is used to explain the result of DNN.
Martínez-velasco et al. ^13^	Age-Related Macular Degeneration and Preeclampsia	Balanced Bagging, BRF, RF, GB, KNN, LR, SVM, DT	Yes	SMOTE and Under-sampling	Balanced bagging and BRF outperform others even in imbalanced datasets.
Agyemang et al. ^14^	Stroke	KNN, SVM, LR, RF, DT	Yes	RO, ADASYN, SMOTE, SMOTE–Tomek	SVM with Random Oversampling performs better.
Abayomi-Alli et al. ^15^	COVID-19	DNN, ExtraTrees, SVM, RBF etc.	Yes	SMOTE	DNN-ExtraTrees ensemble outperforms others.
Elgendy et al. ^16^	Diabetes	LR, RF, MLP, AB, GB etc.	Yes	SMOTE	MLP with Staking gives the highest accuracy.
Dutta et al. ^7^	Diabetes	GNB, BNB, RF, DT, XGB, LGB	Yes	-	DT, RF, XGB, and LGB combination. performance than others.
Alzakari et al. ^17^	Heart Disease	XGB, Bi-LSTM	Yes	-	XGB performs feature selection and early classification, and Bi-LSTM performs second-stage classification.
Das et al. ^18^	Diabetes, Cancer	RF, XGB, AB, CB etc.	Yes	SMOTE	RF performs well in both balanced and imbalance datasets.
Senthilvadivu et al. ^19^	ICU Condition	RF, XGB	Yes	-	XGB performs better than RF.
Abdellatif et al. ^8^	Heart Disease	RF	Yes	SMOTE	Proposed weighted RF performs better than SMOTE-RF.
Yalin et al. ^20^	Diabetes	LR, XGB	Yes	-	The proposed ensemble outperforms other base models.
Abnoosian et al. ^21^	Diabetes	KNN, SVM, DT, RF, AB, GNB	Yes	-	The proposed ensemble outperforms the base models.
Liu et al. ^9^	Cardiovascular disease	LR, SVM, Bagging	Yes	Undersampling	SVM is used as a weak learner for bagging.
Masruriyah et al. ^22^	Heart Disease	C4.5, RF, SVM, LR	Yes	SMOTE, ADASYN	Oversampling decreases the model's performance.
Pablo et al. ^23^	COVID-19	MLP, XGB, NB, DT, SVM	Yes	POS, RUS, SMOTE, ADASYN	MLP stood out strongly for all experimental setups.
Chowdhury et al. ^24^	COVID-19	KNN, SVM, RF, XGB	Yes	SMOTE	The proposed pipeline outperforms other pairs and the base models.
Prithula et al. ^25^	Respiratory diseases	MLP, XGB, DT, SVM, AB CB etc	Yes	SMOTE	CB performs better than the proposed model.
Chowdhury et al. ^26^	Diabetes	LR, RF, AB, GB, Voting	Yes	ENN, SOMTE_N, SMOTE-ENN, SMOTE-Tomek	The performance of the models is better in original dataset.
Mienye and Sun ^10^	Diabetes, Cancer, CKD	LR, DT, XGB, RF	Yes	-	Cost-sensitive XGB performs better than others.

## 3. Proposed methodology

**Figure 1.**
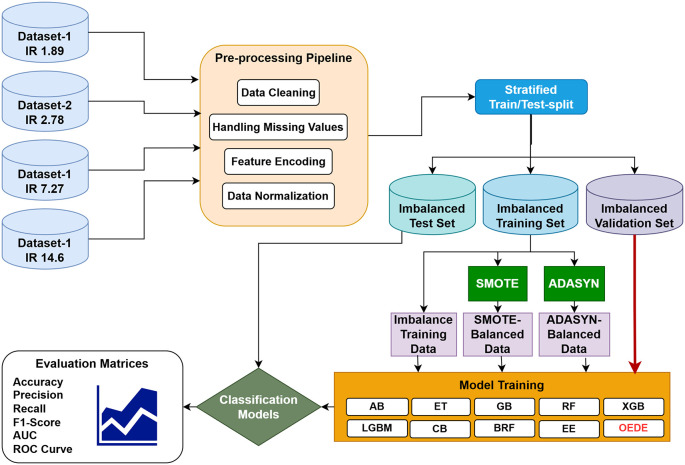
Proposed methodology.

To enhance the prediction accuracy on an imbalanced dataset, we propose a novel ensemble approach called Optimized Ensemble by Differential Evolution (OEDE). An overview of the proposed methodology is shown in Figure 1. Four different medical datasets with different imbalance ratio (1.89 to 14.6) were collected from the UCI Machine Learning Repository to assess the robustness of the model. After data preprocessing, a stratified train-test split was applied to maintain the class distribution in the training, test, and valuation sets. Logistic Regression (LR), Random Forest (RF), and XGBoost (XGB) were used as the base models, and Differential Evolution (DE) was used to combine their predictive strength based on prediction probability on the validation set, with Area Under the ROC curve (AUC) as the optimization objective. Before constructing the final model, the base learners were fine-tuned using GridSearchCV and Stratified-K-Fold cross-validation to ensure a robust model under class imbalance. The performance of OEDE was tested on the original imbalanced dataset, dataset balanced with SMOTE, and ADASYN. A performance comparison was performed against existing ensemble models such as AB, CB, LGBM, ET, EE, and RBF.

### 3.1 Datasets

To evaluate the robustness and adaptability of the proposed OEDE model, we used four widely used public medical datasets from the UCI Machine Learning Repository, with different class Imbalance Ratios (IR), as shown in Table 2. These datasets were carefully chosen to cover a broad range of real-life situations where minority class data are clinically significant. The Pima Indiana Diabetes Dataset (IR = 1.89) includes the inception of diabetes in female patients based on diagnostic measurements. The Haberman’s Cancer Survival Dataset (IR = 2.78) has patient records for those who had undergone breast cancer surgery, aimed at predicting post-operative survival. The Thoracic Surgery Dataset (IR = 7.27) is based on predicting survival following major lung surgery for patients, considering clinical and surgical factors. Finally, the Cervical Cancer Risk Dataset (IR = 14.6) uses personal health information and screening results to assess the risk of cervical cancer. These datasets provide a range of imbalance ratios where the minority class proportion is less than 40%, offering a robust testbed for validating the effectiveness of the OEDE.

**Table 2.** Summary of datasets used.

Dataset name	No. of instances	No. of features	Minority class	Minority class proportion (%)	Imbalance Ratio (IR)
Pima Indians Diabetes ^27^	768	9	Positive	34.9%	1.89
Haberman’s Cancer ^28^	306	4	Negative	26.5%	2.78
Thoracic Surgery ^29^	470	17	Negative	12.1%	7.27
Cervical Cancer Risk ^30^	858	36	Positive	6.4%	14.6

### 3.2 Base models

The proposed ensemble model leverages three different base learners, namely Logistic Regression (LR), Random Forest (RF), and XGBoost (XGB), with the aim of improving predictive performance based on model diversity. The LR is a linear model that predicts the probability of a binary outcome using a sigmoid function. ^31^ Its interpretability and probabilistic output make it a valuable baseline, particularly when modelling linear relationships. ^32, 33^ RF is an ensemble of decision trees that introduces non-linearity and robustness by aggregating predictions from a group of trees trained on bootstrapped datasets and random feature subsets, thus removing variance and overfitting. ^34^ It performed well in capturing complex feature interactions. ^35^ XGBoost builds a sequence of trees, where each tree corrects the errors made by its predecessors, optimizing a normalized objective function *L =* $\sum_{l}l\left(y_{i},\hat{y_{i}}\right)+\sum_{k}\Omega \left(f_{k}\right)$ *,* where $\Omega \left(f\right)=\mathrm{\gamma T}+\frac{1}{2}\lambda {\left\|w\right\|}^{2}.$ ^36^ Its ability to model non-linearities with good precision, ^37^ regularization, and the ability to handle missing values ^20^ makes it a strong learner in the ensemble. Together, these models provide distinct perspectives, such as linear separability, variance minimization, and gradient-based optimization, making the ensemble more robust and less prone to overfitting than any single learner.

### 3.3 Sampling techniques

We used oversampling techniques, the Synthetic Minority Oversampling Technique (SMOTE), and Adaptive Synthetic Sampling (ADASYN) to address the class imbalance of the original dataset. We assessed the performance of our proposed ensemble model on three different datasets: the original imbalanced dataset, SMOTE-balanced dataset, and ADASYN-balanced dataset. SMOTE creates synthetic instances for the minority class through interpolation between minority samples and their k-closest minority neighbour, thereby expanding the decision boundary and reducing overfitting to specific samples. ^38^ While SMOTE assumes the same significance to all instances of the minority class, ^39^ ADASYN focuses on varying the importance of individual minority instances according to their level of difficulty in learning. ^40^ It individually generates a synthetic minority sample, which is harder to classify owing to its lower density in the feature space, thereby promoting the generalization of the challenging part of the data. ^41^ Through this cross-dataset comparison of model performance, we aim to evaluate not only the generalizability of the proposed ensemble, but also how different balancing strategies influence its performance.

### 3.4 OEDE

The Optimized Ensemble by Differential Evolution (OEDE) was designed as a novel ensemble model to alleviate the classification difficulties of imbalanced medical datasets. This method incorporates class-balanced base learners and a Differential Evolution (DE)-driven algorithm for AUC maximization. Three fundamentally different classifiers, LR, RF, and XGB, were used as base models. All base models were configured with a class-weighting mechanism during training to address the effects of class imbalance. LE employs class_weight= ‘balanced’, RF uses class_weight=‘balanced_subsample’, while XGB is fine-tuned for logloss with consideration taken for label imbalance. These learners were independently trained on the same dataset and produced probability estimates for the positive class that were combined using a learned ensemble set of weights. OEDE does not combine the outputs of the base learners using fixed or heuristic-based weights; instead, it uses Differential Evolution (DE) for adaptive weight optimization. DE is a stochastic population-based global optimization method that is well-suited for non-differentiable and non-convex functions. ^42– 45^

Let the prediction probabilities from M base classifiers for a given instance *x* be denoted by [p _1_(x), p _2_(x), …, p _M_(x)]. Assign weight w _i_ to each base classifier *i*, where the weights satisfy:

For the proposed ensemble, assign a weight w _i_ to each base model i such that:

$$\sum_{i=1}^{M}w_{i}=1\;\text{and}\;w_{i}\geq 0\;\text{for all}\;i$$ (1)

The ensemble’s predicted probability is given by the weighted average:

$$p_{\mathrm{ens}}\left(x\right)=\sum_{i=1}^{M}w_{i}p_{i}\left(x\right)$$ (2)

The goal was to choose the weights w = [w _1_, w _2_, …, w _M_] to maximize the AUC on a validation set. Recall that the AUC represents the probability that a randomly chosen positive instance has a higher score than a randomly chosen negative instance.

$$\mathrm{AUC}=\frac{1}{\left|S^{+}\|S^{-}\right|}\sum_{i\in S^{+}}\sum_{j\in S^{-}}I\left(p_{\mathrm{ens}}\left(i\right)>p_{\mathrm{ens}}\left(j\right)\right)$$ (3)

where *S ^+^:* index of positive instances, *S ^-^:* index of negative instances, *I:* indicator function.

Because many optimization algorithms (such as Differential Evolution) are formulated as minimization problems, the loss function is defined as the negative AUC:

$$L\left(w\right)=-\mathrm{AUC}\left(y_{\mathrm{val}},p_{\mathrm{ens}}\left(\mathrm{val}\right)\right)$$ (4)

Thus, the optimization problem becomes:

$$\min_{w\;}L\left(w\right)\;\text{subject to}\;\sum_{i=1}^{M}w_{i}=1,w_{i}\geq 0\forall i$$ (5)

DE is selected for this task because of its robustness in optimizing non-differentiable and non-convex functions such as AUC. DE evolves a population of weight vectors over generations using mutation, crossover, and selection to converge to a globally optimal solution. The proposed process is described by Algorithm 1.

Algorithm 1. **Optimized Ensemble by Differential Evolution (OEDE).**

**Input:**

- Traini Set: X_train, y_train
- Validation Set: X_val, y_val
- Test Set: X_test, y_test
- DE parameters: population size P, generations G

**Output:**

- Test set predictions y_pred
- Evaluation metrics: Accuracy, Precision, Recall, F1-score, AUC

1. Train LR, RF, XGB on (X_train, y_train)

2. For each model m ∈ {LR, RF, XGB}:

3. p_val_m = m.predict_proba(X_val)[:, 1]

4. P_val = [p_val_LR, p_val_RF, p_val_XGB]

5. function AUC_Loss(weights, P_val, y_val):

6. Normalize weights: w = weights/sum (weights)

7. Ensemble prediction: p_ens = dot(P_val, w)

8. Return -AUC(y_val, p_ens)

9. bounds = [(0, 1), (0, 1), (0, 1)]

10. w_opt = DifferentialEvolution (AUC_Loss, bounds, args=(P_val, y_val))

11. w_opt: w_opt = w_opt/sum(w_opt)

12. For each m ∈ {LR, RF, XGB}:

13. p_test_m = m.predict_proba(X_test)[:, 1]

14. P_test = [p_test_LR, p_test_RF, p_test_XGB]

15. p_test_ens = dot(P_test, w_opt)

16. y_pred = 1 if p_test_ens ≥ 0.5 else 0

17. Return y_pred, evaluation metrics

### 3.5 Performance matrices

We used five common yet important classification metrics such as Accuracy, Precision, Recall, F1-Score, and Area Under the ROC curve (AUC), to evaluate the performance of the proposed OEDE model. While working with the imbalanced dataset, these measures offer a comprehensive insight into the efficiency of the model. Let P _C_, N _C_, P _E_, and N _E_ represent the numbers of correctly classified positives, correctly classified negatives, false positives, and false negatives, respectively.

• The model’s overall correctness is gauged by accuracy.

$$\text{Accuracy}=\frac{P_{c}+N_{E}}{P_{c}+N_{C}+P_{E}+N_{E}}$$ (6)

• The percentage of correct positive predictions among all positive predictions is known as the precision.

$$\text{Precision}=\frac{P_{c}}{P_{c}+P_{E}}$$ (7)

• Recall shows how well the model recognizes the actual positives.

$$\text{Recall}=\frac{P_{c}}{P_{c}+N_{E}}$$ (8)

• F1-Score balances the Precision and Recall.

$$F1=2\times \frac{\text{Precision}\times \text{Recall}}{\text{Precision}+\text{Recall}}$$ (9)

When it comes to an imbalanced dataset, the AUC is especially significant, which represents the area under the ROC curve by plotting the true positive rate $\left(\frac{P_{C}}{P_{c}+N_{E}}\right)$ and false positive rate $\left(\frac{P_{E}}{P_{E}+N_{C}}\right)$ across different classification thresholds. A higher AUC represents better separability. Finally, we visualized the ROC curve of each model, which offered an intuitive view of the trade-off between false alarms and sensitivity. The closer the curve is to the upper-left corner, the better is the classifier.

## 4. Results and discussion

The experiment involved four different medical datasets with different imbalance ratios (1.89 – 14.6) used for the performance analysis of the proposed model. This section provides a detailed analysis of the proposed model and compares the performance matrices with the existing base models after training them in three different conditions: the original imbalanced dataset, data balanced using SMOTE, and data balanced using ADASYN. The performance matrices were evaluated using a never-observed imbalance test split.

### 4.1 Pima indiana diabetes dataset

**Figure 2.**
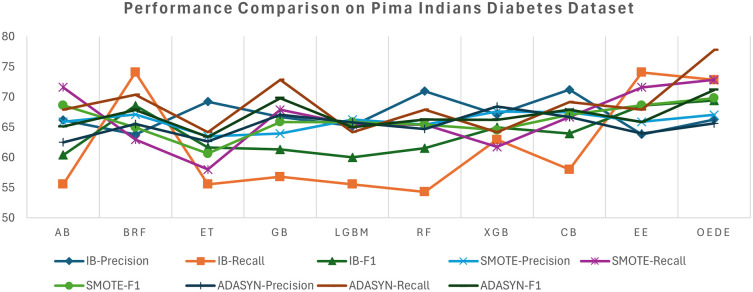
Precision, Recall, and F1-score comprise between OEDE and state-of-the-art ML models on the Pima Indiana Diabetes Dataset.

On the original dataset, OEDE achieved high accuracy and AUC score, as shown in Table 3, effectively discriminating between diabetic and non-diabetic samples without artificial rebalancing. Although ensemble models such as RF and XGBoost demonstrated competitive accuracy, their AUC and recall values were low, indicating a bias towards the majority classes. The OEDE tended to have a high F1 score, while maintaining a better balance between precision and recall, as shown in Figure 2. The overall performance of all models improved when the dataset was balanced using SMOTE, as indicated by the increased recall and F1-score, but OEDE still outperformed the others in terms of accuracy, AUC, and F1-score. Notably, the change in the AUC value was marginal, indicating that merely balancing the data may not be sufficient. In the ADASYN-balanced scenario, it tends to echo those of SMOTE but with minor instability for some models because ADASYN introduces noise in the minority samples. OEDE again outperformed other baseline models with a minor drop in precision while maintaining the AUC and showed a balanced performance throughout the datasets.

**Table 3.** AUC and Accuracy comparison of OEDE and state-of-the-art ML models on Pima Indiana diabetes dataset.

Models	Original imbalanced data		SMOTE-Balanced data		ADASYN-Balanced data	
	Accuracy	AUC	Accuracy	AUC	Accuracy	AUC
AB	74.46	80.1	77.06	82.4	74.46	80.44
BRF	76.19	83.9	76.19	83.49	76.62	84.17
ET	75.76	82.4	73.59	81.79	74.03	81.5
GB	74.89	82.77	75.32	84.26	77.92	82.94
LGBM	74.03	81.52	76.19	80.98	75.76	81.81
RF	76.19	83.15	75.76	85.35	75.76	84.16
XGB	76.19	81.33	76.19	80.89	77.06	80.87
CB	77.06	83.82	77.06	83.93	77.06	82.45
EE	76.19	79.81	77.06	82.4	75.32	80.05
**OEDE**	**77.49**	**84.02**	**77.92**	**84.31**	**77.92**	**84.37**

### 4.2 Haberman’s cancer dataset

**Figure 3.**
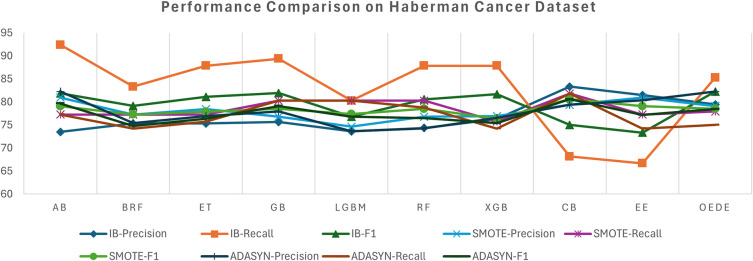
Precision, Recall, and F1-score comprise between OEDE and state-of-the-art ML models on Haberman’s Cancer Dataset.

OEDE achieved a higher accuracy, AUC, and F1-score than traditional ensemble models such as RF, AB, and CB in an imbalanced dataset, as shown in Table 4. While some models show comparable accuracy, OEDE shows stability in precision and recall, leading to a better F1-score as shown in Figure 3, demonstrating its ability to detect the minority class without compromising the overall correctness. After the dataset is balanced with SMOTE, an improvement is observed in recall for most of the models, which shows that the models benefit from the synthetic sample. The OEDE maintained its AUC lead, highlighting its ability to influence informative patterns more effectively, even in a balanced dataset. Its high F1-score reflects food robustness against overfeeding for synthetic data. When ADASYN is used for data balancing, some models show instability in precision and recall, as ADASYN tends to produce harder-to-learn synthetic samples, while OEDE has a high-performance score without sacrificing stability and sensitivity.

**Table 4.** AUC and Accuracy comparison of OEDE and state-of-the-art ML models on Haberman’s Cancer Dataset.

Models	Original imbalanced data		SMOTE-Balanced data		ADASYN-Balanced data	
	Accuracy	AUC	Accuracy	AUC	Accuracy	AUC
AB	70.65	61.04	70.65	66.75	71.74	67.74
BRF	68.48	62.88	67.39	61.68	64.13	61.8
ET	70.65	60.61	68.48	64.22	66.3	64.66
GB	71.74	68.76	68.48	67.45	69.57	67.4
LGBM	65.22	65.33	66.3	61.77	65.22	62.47
RF	69.57	66.03	68.48	66.08	65.22	66.9
XGB	71.74	67.77	66.3	66.61	65.22	66.14
CB	67.39	70.8	71.74	66.96	71.74	67.74
EE	65.22	68.12	70.65	66.75	68.48	66.78
**OEDE**	**72.83**	**73.25**	**68.48**	**70.37**	**69.57**	**66.33**

### 4.3 Thoracic surgery data

**Figure 4.**
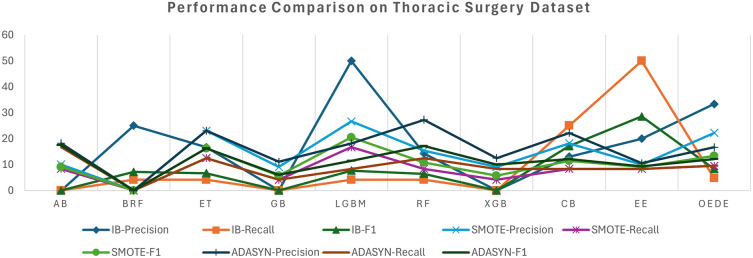
Precision, Recall, and F1-score comprise between OEDE and state-of-the-art ML models on the Thoracic surgery dataset.

The thoracic surgery dataset showed a significant imbalance in class distribution, challenging most of the classifiers that favour the majority classes. OEDE outperformed traditional ensemble models such as RF, LGBM, and BRF in terms of AUC and F1-score, as shown in Table 5 and Figure 4, while the original imbalanced dataset was used. Although many baseline models achieve relatively high accuracy, OEDE maintains its balanced performance, achieving higher recall for minority classes and offering better overall performance. With the SMOTE-balanced dataset, most models improve recall values owing to synthetic samples. OEDE continues to display a superior performance matrix, especially the AUC value, which indicates the robustness of the model even when the dataset is balanced with synthetic data without dropping precision or overfitting. Similar to the previous datasets, the performance of the baseline models fluctuated in the ADASYN-balanced dataset, but OEDE maintained a stable performance and showed high F1 and AUC scores. This generalizability and consistent performance are possible owing to the differential evaluation-based weight optimization.

**Table 5.** AUC and Accuracy comparison of OEDE and state-of-the-art ML models on the Thoracic Surgery dataset.

Models	Original imbalanced data		SMOTE-Balanced data		ADASYN-Balanced data	
	Accuracy	AUC	Accuracy	AUC	Accuracy	AUC
AB	80.14	47.15	71.63	44.14	73.05	50.11
BRF	81.56	50.48	77.31	47.61	75.89	46.19
ET	80.14	48.11	78.01	50.89	78.01	45.76
GB	82.98	56.29	76.6	54.93	78.01	50.04
LGBM	82.98	50.57	78.01	53.45	78.01	50.18
RF	79.43	50.82	76.6	47.86	79.43	48.15
XGB	82.98	50.82	76.6	44.23	74.47	43.38
CB	58.87	49.61	78.01	52.62	79.43	49.73
EE	57.45	57.59	71.63	44.14	72.34	47.19
**OEDE**	**84.4**	**70.08**	**81.56**	**69.37**	**79.43**	**65.4**

### 4.4 Cervical cancer risk dataset

**Figure 5.**
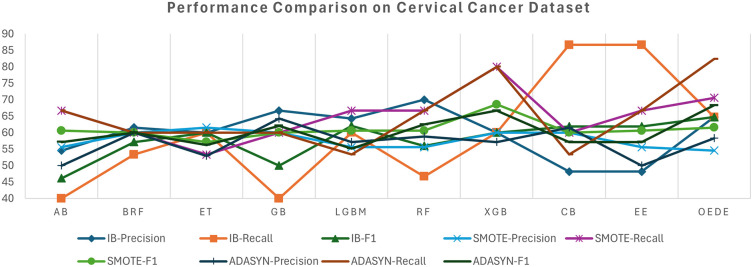
Precision, Recall, and F1-score comprise between OEDE and state-of-the-art ML models on the Cervical Cancer Risk Dataset.

The Cervical Cancer Risk dataset shows a significant difficulty owing to high-class imbalance, where high-risk cases are less than 7% of the dataset, which tends to affect the performance of the traditional model. On the imbalanced dataset, most baseline models, such as RF, ET, and CB, struggle with the minority class and show a low recall and F1-score, despite considerable accuracy, as shown in Table 6 and Figure 5. In contrast, OEDE is able to distinguish the minority class effectively owing to adaptive weighting, as reflected in the AUC and F1-score. Again, SMOTE improves the recall for all models, including OEDE, while maintaining competitive precision and achieving the highest AUC. For the ADASYN-balanced dataset, OEDE maintained high performance with the highest AUC and F1-score, supporting its resilience and generalizability.

**Table 6.** AUC and Accuracy comparison of OEDE and state-of-the-art ML models on the cervical cancer risk dataset.

Models	Original imbalanced data		SMOTE-Balanced data		ADASYN-Balanced data	
	Accuracy	AUC	Accuracy	AUC	Accuracy	AUC
AB	93.57	81.45	94.96	89.22	94.19	92.24
BRF	94.35	94.94	95.35	96.3	94.35	95.47
ET	94.35	92.27	95.35	89.33	94.57	88.31
GB	94.35	94.13	95.35	96.38	94.74	95.02
LGBM	94.74	94.05	94.96	94.29	94.96	93.03
RF	94.74	94.27	94.96	94.6	94.35	94.05
XGB	94.35	93.42	94.74	96.21	94.35	96.08
CB	92.8	95.17	94.35	97.23	94.35	96.89
EE	92.8	95.12	94.96	89.22	94.19	92.24
**OEDE**	**95.35**	**96.73**	**95.19**	**97.19**	**94.96**	**97.89**

**Figure 6.**
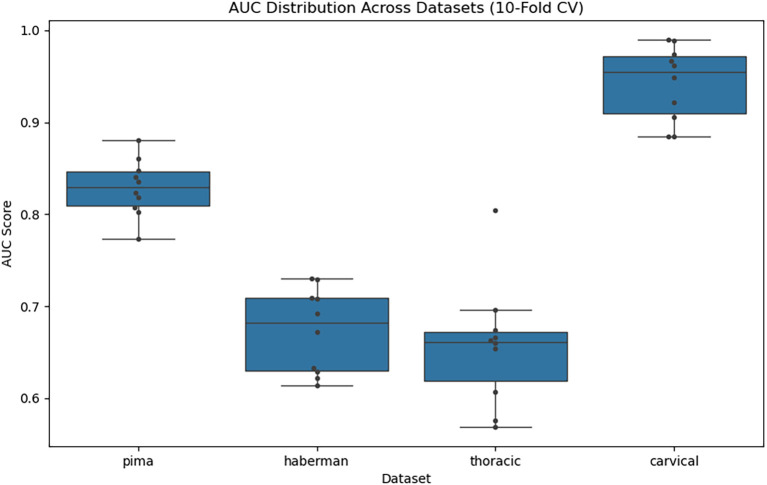
AUC distribution across datasets.

The box plot overlaid with swarm points ( Figure 6) demonstrates the diversity of the different datasets. The median and IQR of AUC on the Cervical dataset presented the highest AUC scores (~0.95 median), demonstrating excellent and stable classification performance. Even for the Pima dataset, a moderate spread was observed, indicating reasonable generalization for the model. The Haberman and Thoracic datasets, on the other hand, demonstrated lower and more variable AUCs, around a median of 0.66–0.68, suggesting struggles possibly with class imbalance or limited separability.

**Figure 7.**
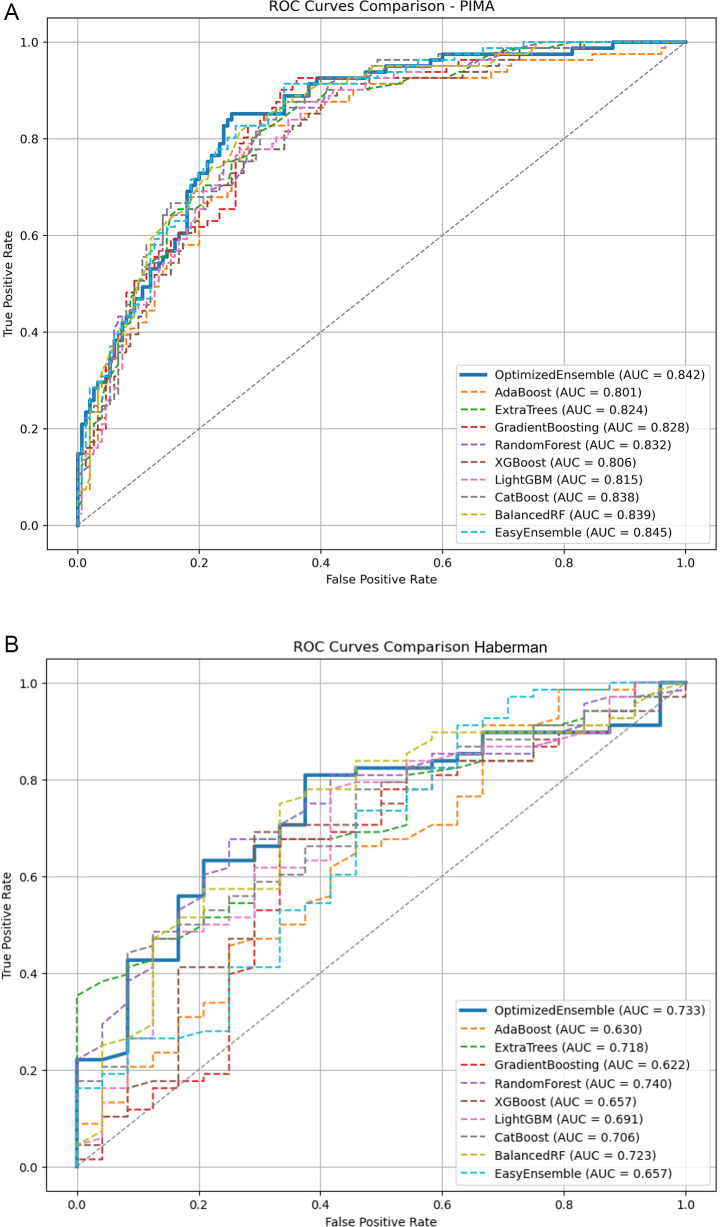

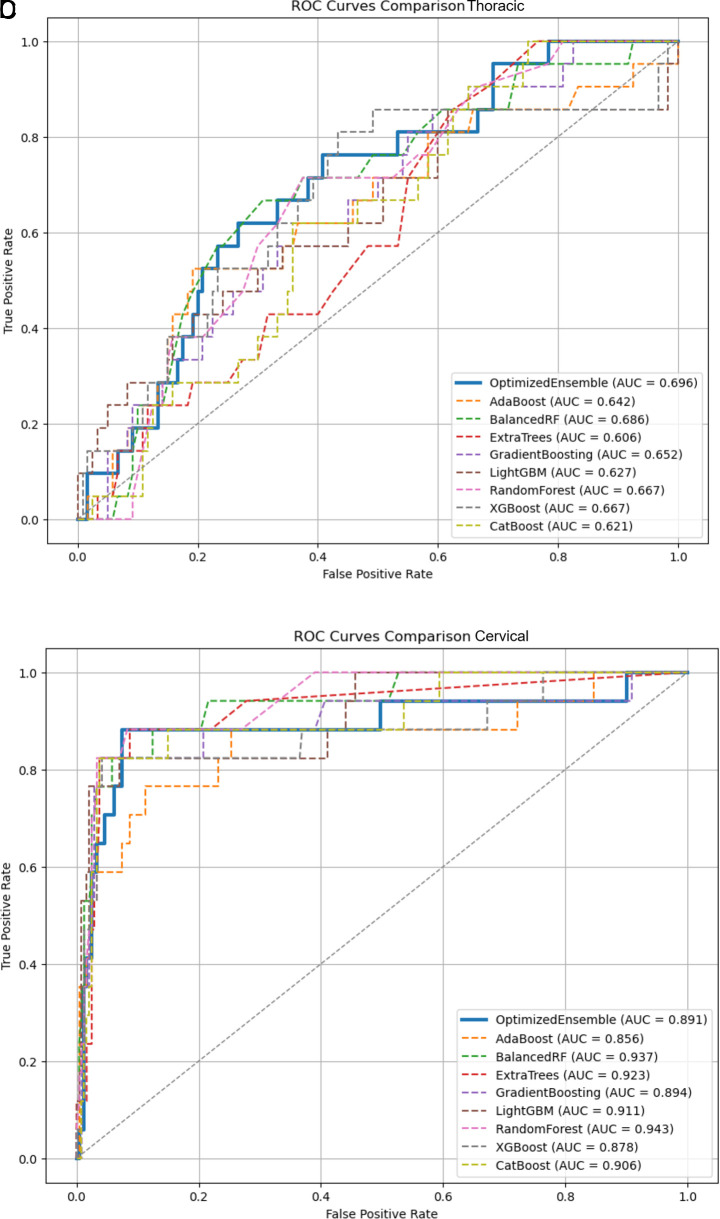
ROC plots of all the models on different datasets. **(A)** Pima Indiana Diabetes Dataset. **(B)** Haberman’s Cancer Dataset. **(C)** Thoracic Surgery Dataset. **(D)** Cervical Cancer Risk Dataset.

The performance of the OEDE across the four benchmark datasets demonstrates its robustness and adaptability across balanced and imbalanced datasets. Despite varying levels of class imbalance, OEDE achieved superior performance, outperforming a range of traditional baseline models. To support the numerical findings, ROC curves were plotted ( Figure 7) for all the datasets to visually represent the discrimination ability of the model. Consistently across all datasets, the ROC curve of OEDE was favourable, aligning with the AUC values achieved.

## 5. Conclusion

In this study, a novel ensemble model, OEDE, was proposed to address a methodological gap in medical classification and the challenges of imbalanced medical datasets. The limitation of traditional ensemble techniques that rely on static weight initialization and surrogate loss function thus fails to optimize non-differentiable clinically relevant matrices. The key innovation includes the ensemble integration framework of the predictive power of diverse classifiers such as LR, RF, and XGB through a differential evolution-based ensemble, which learns the optimal weight by maximizing the AUC. Unlike traditional models, OEDE adapts the decision boundary to enhance the discrimination capacity, especially in the minority class. The approach also includes class-balanced-based learning, ensuring that base models reduce the imbalance at the source, resulting in a robust ensemble model that generalizes well across datasets with different characteristics and imbalance ratios. The performance of OEDE was assessed on four different medical datasets with class imbalance ratios from 1.89 to 14.6, to verify its ability to handle class imbalance data. The results demonstrated that OEDE almost always performed significantly better than the state-of-the-art ML models in terms of AUC, accuracy, and F1-score, and the model was robust under different data balancing techniques such as SMOTE and ADSYN. Adding the ROC curves of all the models and datasets also confirms the superior separability of OEDE and makes it a useful framework for practical real-world classification problems.

## Ethical approval

This study does not require any ethical approval since this study only used publicly accessible, de-identified datasets. There were no experiments with human subjects, and no new data were gathered. The datasets do not contain any personally identifiable information and are available from recognized open repositories.

## Data Availability

The datasets used in this research are publicly available from recognized data repositories and can be accessed through the following links. The Pima Indians Diabetes Dataset, originally hosted on UCI ML Repository is no longer available there. However, it can be accessed via Mendeley Data.
• UCI Machine Learning Repository. Haberman’s Survival Dataset. DOI: 10.24432/C5XK51
• UCI Machine Learning Repository. Thoracic Surgery Data. DOI: 10.24432/C5Z60N
• UCI Machine Learning Repository. Cervical Cancer Dataset. DOI: 10.24432/C5Z310
• Mendeley Data. Pima Indians Diabetes Dataset. DOI: 10.17632/7zcc8v6hvp.1 All the data is publicly available under the terms of the **Creative Commons Attribution 4.0 International** **(CC BY 4.0)**.
