# Supplementary material for: A Differential Evolution-Based Optimized Ensemble for Balanced and Imbalanced Medical Datasets
Source: F1000Res. 2026 Jan 27;14:1003. Originally published 2025 Sep 29. [Version 2] doi: 10.12688/f1000research.169456.2 (PMC12895097; doi:10.12688/f1000research.169456.2)
Supplement: Peer review history

# Reviewer report: Reviewer response for version 2

**DOI:** 10.5256/f1000research.195381.r453315

**Published:** 2026-02-11

**Authors:** Charles Ikerionwu

**Affiliations:** 1 Federal University of Technology, Owerri, Nigeria

Having effected the corrections, the author's paper can be accepted in its current form.

Is the work clearly and accurately presented and does it cite the current literature?

Yes

If applicable, is the statistical analysis and its interpretation appropriate?

Partly

Are all the source data underlying the results available to ensure full reproducibility?

Yes

Is the study design appropriate and is the work technically sound?

Yes

Are the conclusions drawn adequately supported by the results?

No

Are sufficient details of methods and analysis provided to allow replication by others?

Partly

Reviewer Expertise:

Application of AI/ML in multidisciplinary research such as agriculture, software engineering, health and energy. Software process improvement and data science.

I confirm that I have read this submission and believe that I have an appropriate level of expertise to confirm that it is of an acceptable scientific standard.

# Reviewer report: Reviewer response for version 1

**DOI:** 10.5256/f1000research.186799.r443187

**Published:** 2026-01-05

**Authors:** Charles Ikerionwu

**Affiliations:** 1 Federal University of Technology, Owerri, Nigeria

Abstract: 

 Improve the quantification of the result.

 Body of the work:

 Numbering of Equation is preferably done on the right-hand side.

 The authors appear to establish a gap using this sentence: 

 "Some of these studies enhanced the performance to some extent, but they were not flexible in assigning weights based on the weights and did not directly optimize the corresponding relevant evaluation measure." However, the sentence could not clearly establish the gap the study is pursuing. 

 Supporting the gap with a citation would present the gap clearly.

 What type of gap has been identified? - methodology, knowledge, etc. In the conclusion, no reference was made to the findings and how it closed the gap. 

 In addressing imbalance dataset, SMOTE and ADSYN have been used in earlier research, yet this study claims it is novel. 

 The use of Logistic Regression (LR), Random Forest (RF), and XGBoost (XGB) has been adopted in earlier research. The same is DE. 

 Thus, the main contributions of this study as listed in section 1 should be revisited. 

Is the work clearly and accurately presented and does it cite the current literature?

Yes

If applicable, is the statistical analysis and its interpretation appropriate?

Partly

Are all the source data underlying the results available to ensure full reproducibility?

Yes

Is the study design appropriate and is the work technically sound?

Yes

Are the conclusions drawn adequately supported by the results?

No

Are sufficient details of methods and analysis provided to allow replication by others?

Partly

Reviewer Expertise:

Application of AI in multidisciplinary research such as agriculture, software engineering, health and energy. Software process improvement and data science.

I confirm that I have read this submission and believe that I have an appropriate level of expertise to confirm that it is of an acceptable scientific standard, however I have significant reservations, as outlined above.

# Response

**Published:** 2026-01-16

**Authors:** Biswajit Sahoo

**Affiliations:** School of Computer Engineering, Kalinga Institute of Industrial Technology, Bhubaneswar, Odisha, India

We would like to thank the reviewer for their detailed and constructive feedback. The comments regarding the quantification of results, the definition of the research gap, and the clarification of our novel contributions were particularly helpful. We have carefully revised the manuscript to address these points. Below, we provide a point-by-point response to each comment.

**Comment 1:** *"Improve the quantification of the result."*

 We appreciate this suggestion. We have revised the abstract to replace qualitative statements (e.g., "consistently outperforms") with specific, quantified evidence from our experiments. Specifically, we have highlighted the significant performance margin achieved on the most difficult dataset (Thoracic) and the peak performance on the best-performing dataset (Cervical Cancer).

**Comment 2:** *"Numbering of Equation is preferably done on the right-hand side."*

 Thank you for pointing this out. We originally formatted the manuscript with standard right-aligned equation numbering. The current layout (with numbers appearing on a separate line or to the left) was introduced during the journal's typesetting and production process for the published version.

 In the revised version (Version 2), we will explicitly request the F1000Research production team to correct the alignment to ensure equation numbers appear on the right-hand side, consistent with standard mathematical formatting.

**Comment 3:** *"The authors appear to establish a gap using this sentence... However, the sentence could not clearly establish the gap the study is pursuing. Supporting the gap with a citation would present the gap clearly."*

 We acknowledge that the original gap analysis was vague. We have rewritten this section to explicitly contrast the limitation of "static" ensemble weights and "surrogate loss functions" (used in standard methods) against our proposed method. We have also added citations to support the claim that standard gradient-based methods cannot directly optimize non-differentiable metrics like AUC.

**Comment 4:** *"What type of gap has been identified? - methodology, knowledge, etc. In the conclusion, no reference was made to the findings and how it closed the gap."*

 We have identified this as a **Methodological Gap**. The existing methods utilize optimization techniques that require differentiable functions, preventing them from optimizing the AUC directly. We have revised the conclusion to explicitly state this gap type and provided specific reference to our findings (specifically the Thoracic dataset results) to demonstrate how our methodology successfully closed this gap where traditional models failed.

**Comment 5:** *"In addressing an imbalanced dataset, SMOTE and ADSYN have been used in earlier research, yet this study claims it is novel."*

 We agree with the reviewer that SMOTE and ADASYN are well-established techniques. We have clarified in the manuscript that we do not claim the *use* of SMOTE/ADASYN as the primary novelty. Rather, these are employed as necessary preprocessing steps to ensure the base learners are competent. The novelty lies in the **ensemble integration framework** that optimizes the combination of these preprocessed learners.

**Comment 6:** *"The use of Logistic Regression (LR), Random Forest (RF), and XGBoost (XGB) has been adopted in earlier research. The same is DE."*

 This is a crucial observation. While the individual components (LR, RF, XGB, DE) are indeed established, the **architectural integration** proposed in OEDE is novel. Standard stacking ensembles use a meta-learner (like Logistic Regression) that minimizes error via Gradient Descent. Because AUC is non-differentiable, standard stacking cannot optimize AUC directly.

 Our contribution is the **methodological innovation** of replacing the standard meta-learner with a Differential Evolution optimizer. This allows the ensemble to solve the "weights assignment" problem as a global optimization task, directly maximizing AUC. This specific integration addresses the methodological gap described in Comment 4.

**Comment 7:** *"Thus, the main contributions of this study as listed in section 1 should be revisited."*

 In light of the feedback regarding novelty (Comments 5 and 6), we have rewritten the contributions section to focus on the **integration strategy** and the **direct optimization capability**, rather than simply listing the algorithms used.

# Reviewer report: Reviewer response for version 1

**DOI:** 10.5256/f1000research.186799.r428576

**Published:** 2025-11-22

**Authors:** Najah Al-shanableh

**Affiliations:** 1 Al al-Bayt University, Mafraq, Jordan

The assessment of the submitted work indicates that it meets all key criteria for clarity, rigor, transparency, and reproducibility. The presentation of the work is clear, accurate, and well-structured, with appropriate citations to current and relevant literature. This demonstrates a solid understanding of the field and ensures that the study is well-grounded in existing research.

 The study design is appropriate for the stated objectives, and the technical execution appears sound. The methodological steps and analytical procedures are described in sufficient detail to allow independent replication by other researchers, which strengthens the credibility and scientific value of the work.

 Overall, the work demonstrates strong methodological rigor, clarity of presentation, and adherence to good scientific practices.

Is the work clearly and accurately presented and does it cite the current literature?

Yes

If applicable, is the statistical analysis and its interpretation appropriate?

Not applicable

Are all the source data underlying the results available to ensure full reproducibility?

Yes

Is the study design appropriate and is the work technically sound?

Yes

Are the conclusions drawn adequately supported by the results?

Yes

Are sufficient details of methods and analysis provided to allow replication by others?

Yes

Reviewer Expertise:

My research focuses on advancing data-driven solutions at the intersection of artificial intelligence, machine learning, data mining, and healthcare analytics.

I confirm that I have read this submission and believe that I have an appropriate level of expertise to confirm that it is of an acceptable scientific standard.

## References

[1] : Advanced Ensemble Machine Learning Techniques for Optimizing Diabetes Mellitus Prognostication: A Detailed Examination of Hospital Data. *Data and Metadata* .2024;3: 10.56294/dm2024.363 10.56294/dm2024.363
